# The site of allergen expression in hematopoietic cells determines the degree and quality of tolerance induced through molecular chimerism

**DOI:** 10.1002/eji.201243277

**Published:** 2013-06-14

**Authors:** Ulrike Baranyi, Martina Gattringer, Andreas M Farkas, Karin Hock, Nina Pilat, John Iacomini, Rudolf Valenta, Thomas Wekerle

**Affiliations:** 1 Division of Transplantation, Department of Surgery, Medical University of ViennaVienna, Austria; 2 Renal Division, Transplantation Research Center, Brigham and Women's Hospital and Children's Hospital, Harvard Medical SchoolBoston, MA, USA; 3 Division of Immunopathology, Department of Pathophysiology and Allergy Research, Center of Physiology and Pathophysiology, Infectiology and Immunology, Medical University of ViennaVienna, Austria

**Keywords:** Allergy, B-cell tolerance, Molecular chimerism, Phl p 5, T-cell tolerance

## Abstract

The transplantation of allergens (e.g. Phl p 5 or Bet v 1) expressed on BM cells as membrane-anchored full-length proteins leads to permanent tolerance at the T-cell, B-cell, and effector-cell levels. Since the exposure of complete allergens bears the risk of inducing anaphylaxis, we investigated here whether expression of Phl p 5 in the cytoplasm (rather than on the cell surface) is sufficient for tolerance induction. Transplantation of BALB/c BM retrovirally transduced to express Phl p 5 in the cytoplasm led to stable and durable molecular chimerism in syngeneic recipients (∼20% chimerism at 6 months). Chimeras showed allergen-specific T-cell hyporesponsiveness. Further, Phl p 5-specific T_H_1-dependent humoral responses were tolerized in several chimeras. Surprisingly, Phl p 5-specific IgE and IgG_1_ levels were significantly reduced but still detectable in sera of chimeric mice, indicating incomplete B-cell tolerance. No Phl p 5-specific sIgM developed in cytoplasmic chimeras, which is in marked contrast to mice transplanted with BM expressing membrane-anchored Phl p 5. Thus, the expression site of the allergen substantially influences the degree and quality of tolerance achieved with molecular chimerism in IgE-mediated allergy.

## Introduction

The induction of tolerance to prevent or treat IgE-mediated allergies is a major goal, since allergic diseases are increasing health concerns in industrialized nations. Therapeutic induction of tolerance toward allergens can be achieved by allergen-specific immunotherapy [1, 2]. Subcutaneous injection immunotherapy applied repeatedly in gradually increasing doses of allergens can alter cellular and humoral allergen-specific immune responses. The inhibition of allergic inflammation by induction of allergen-specific blocking IgG and T-cell tolerance seem to be mechanisms how subcutaneous injection immunotherapy modulates the immune system [1–4]. However, allergen-specific immunotherapy has a number of drawbacks requiring many treatments over a long period of time and pose a significant risk of severe reactions such as anaphylaxis [5]. Cell-based therapies are promising strategies to induce tolerance in several immunological disorders such as autoimmune diseases and organ transplantation [6–8]. Molecular chimerism is one cell-based therapy approach based on the transplantation of autologous (i.e. syngeneic in experimental models) HSCs that have been modified ex vivo to express disease-causing Ags [9]. The state of co-expression of nonmodified and exogenous Ag-expressing hematopoietic cells (i.e. molecular chimerism) has been demonstrated to induce robust and Ag-specific long-term tolerance in some autoimmune disorders and transplantation in preclinical studies [10–12]. Molecular chimerism studies have been demonstrated to induce T-cell and B-cell tolerance toward different surface Ags (e.g. membrane-bound MHC molecules or MOG) and secreted Ags (e.g. proinsulin II) [13–19].

Recently we showed that expression of major allergens, namely the grass pollen allergen Phl p 5 (Phleum pratense) and the birch pollen allergen Bet v 1 (Betula verrucosa), induce robust and long-lasting tolerance toward the allergen in the peripheral blood of myeloablatively and nonmyeloablatively conditioned recipients. Persistent molecular macro- and microchimerism induces long-term tolerance toward allergens that have been introduced into HSCs as surface proteins [20–22]. In prophylactic approaches, complete prevention of Phl p 5-specific IgE and IgG responses could be demonstrated [20]. Besides, T-cell tolerance and tolerance at the effector-cell level were established in these studies.

With regard to translating this molecular chimerism strategy to a therapeutic approach, the surface expression of complete allergens entails the risk of inducing significant side effects (i.e. anaphylaxis). The allergenic activity of allergens is mediated by the number of B-cell epitopes to which IgE can bind and by the number of T-cell epitopes capable of inducing T_H_2 responses [23]. Subsequently, the specific recognition of IgE epitopes of the allergen by mast cell bound IgE-antibodies is responsible for development of immediate allergic inflammation [1]. The presentation of complete allergens on the surface of cells could lead to mediator release in presensitized individuals in cell-based approaches [9]. The use of hypoallergenic allergen derivatives or alternatively intracellular expression of allergens could potentially avoid this risk.

Cytosolic proteins are presented via MHC class I to CD8^+^ T cells after degradation of the proteins by the proteasome. Such proteins are additionally presented by MHC class II if they are degraded through autophagy or TAP-independent pathways [24–27]. It has been postulated for MHC molecules that surface expression is necessary to establish central T-cell tolerance [28]. Furthermore, regulatory T cells were found to be sufficient to control alloantigen-specific (i.e. T-cell-dependent Ags) B-cell responses by suppressing T-cell help in a model of heart transplantation using costimulation blockade and donor-specific transfusion [29]. In contrast, cytosolic expression appears to suffice for non-MHC Ags. [30]. In a “congeneic” model using cytosolic GFP as the only differing Ag, chimeric mice became tolerant toward skin grafts (a stringent tolerance test in allotransplantation, and did not develop humoral responses [30, 31]. Thus, T-cell tolerance toward some, but not all, T-cell-dependent Ags is sufficient to induce B-cell tolerance when the Ag is expressed in the cytoplasm [29].

Here, we investigated the type and degree of tolerance that can be achieved by the expression of a clinically relevant respiratory allergen, Phl p 5, which contains both T- and B-cell epitopes.

## Results

### Cytoplasmic expression of Phl p 5 in mammalian cells

**Figure 1:**
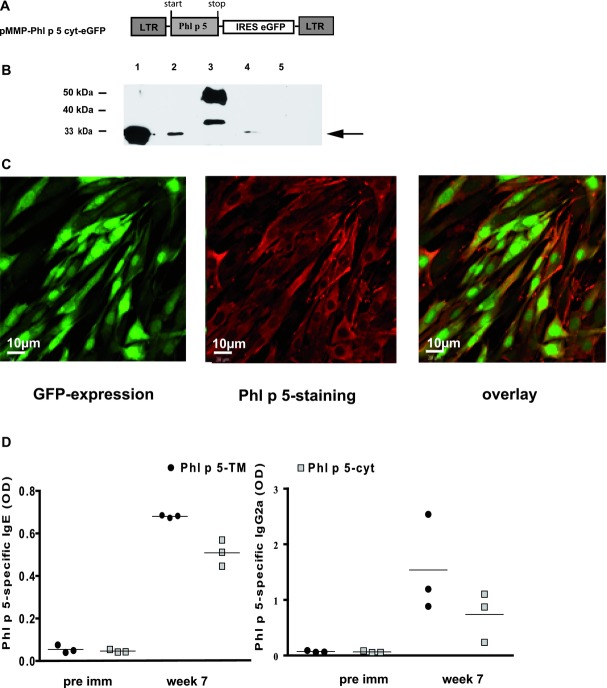
Expression of complete Phl p 5 in the cytoplasm after transduction with a retroviral construct. (A) Schematic representation of the plasmid pMMP-Phl p 5 cyt-eGFP. Long terminal repeats (LTRs) and internal ribosomal entry site (IRES) eGFP expression are indicated. (B) Autoradiography of an immunoblot showing the specific expression of Phl p 5 in lysates of VSV-Phl p 5-cyt transduced 293 T cells (indicated by an arrow, lane 1), rPhl p 5 (lanes 2, 4) surface expressed Phl p 5 in lysates of VSV-Phl p 5-TM transduced 293 T cells (lane 3), and rBet v 1 as a negative control (lane 5). Expression was detected by Phl p 5-specific antibodies. (C) Confocal microscopy of GFP expression in VSV-Phl p 5 cyt transduced cells (left), Phl p 5 was detected exclusively in the cytoplasm of transduced 293 T cells (middle). An overlay of the left and the middle images is shown (right). Images were taken at 40× magnification. (D) Phl p 5-specific IgE and IgG_2a_ levels in the sera of BALB/c mice (*n* = 3) immunized with 3T3 cells transduced with retroviruses to express Phl p 5 on the surface (Phl p 5-TM) or in the cytoplasm (Phl p 5-cyt). Bars represent means. Data shown are from one experiment representative of two experiments performed.

To introduce the full-length allergen Phl p 5 into the cytoplasm in HSC, we generated the retroviral vector pMMP-Phl p 5 cyt-eGFP (Fig. 1A) co-expressing the reporter protein GFP by an internal ribosomal entry site (IRES). GFP was demonstrated to be nontoxic when transduced into HSCs [32]. Recombinant retroviruses were produced in 293 T cells resulting in VSV-Phl p 5-cyt. The expression of complete Phl p 5 was confirmed in lysates of infected 293 T cells in Phl p 5-specific immunoblot assay (Fig. 1B) showing the same molecular weight like the recombinant (r) Phl p 5. The membrane-anchored version shows a higher band (due to the additional transmembrane domain) plus an additional prominent band possibly due to a posttranslational modification [9]. Further, Phl p 5 and GFP co-localization in the cytoplasm of mammalian cells was demonstrated by confocal microscopy of VSV-Phl p 5 cyt transduced murine cells (NIH 3T3 cells; Fig. 1C). Cells transduced with VSV-Phl p 5-cyt did not show expression of Phl p 5 on the surface of 3T3 cells detectable by flow cytometry (FCM), which is in contrast to 3T3 cells transduced with VSV-Phl p 5-TM (the membrane-anchored version; Supporting Information Fig. 1). These data demonstrate that transduction with the construct Phl p 5 allows expression in full length in the cytoplasm. To investigate whether Ag-presentation of the cytosolic Phl p 5 results in effective humoral sensitization, mice were subcutaneously immunized with intact mouse fibroblasts transduced with either membrane-anchored or cytosolic-expressing Phl p 5. Fibroblasts expressing Phl p 5 either on the surface or in the cytoplasm induced specific Ab levels (IgG and IgE; Fig. 1D). Thus, presentation of the cytoplasmic allergen via MHCII molecules induces allergen-specific Ab production.

### Transplanted VSV-cyt-transduced HSCs induce long-term molecular macrochimerism

**Figure 2:**
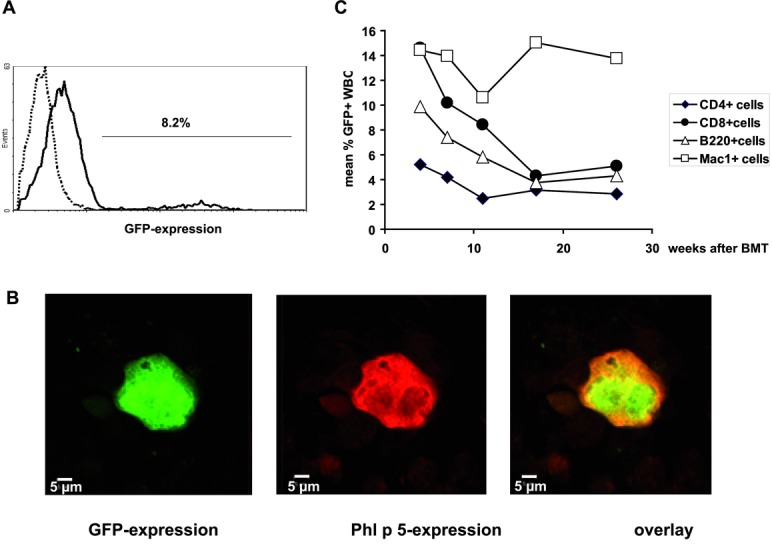
Long-term molecular macrochimerism of Phl p 5-cyt in recipients of VSV-Phl p 5-cyt transduced syngeneic BM. (A) Flow cytometric analysis of GFP expression in transduced BM cells. Black line histogram represents GFP expression in BM of BALB/c mice transduced with VSV-Phl p 5-cyt before BMT. Dotted line histogram represents nontransduced BM cells. (B) Expression of GFP (left) by confocal microscopy of transduced BM cell. Phl p 5 expression was confirmed in the same cells (middle) and an overlay (right). Images were taken at 40× magnification. (C) Expression of GFP within BM-transplanted recipients. The mean percentage of GFP-expressing CD4^+^ cells (black diamonds), CD8^+^ cells (black circles), B220^+^ cells (open triangles), and myeloid cells (open squares) is shown at several time points until week 28 (*n* = 10). Data are shown for one of two representative experiments performed.

Phl p 5 was integrated into BM cells (BMCs) by retroviral transduction of VSV-Phl p 5-cyt. The transduction efficiency was determined by FCM (8.2and 7.1% in twoindependent experiments; Fig. 2A). Further, Phl p 5 and GFP co-expression in BMCs were confirmed by immunofluorescence (Fig. 2B). Transduced BMCs (7 × 10^6^) were transplanted into preconditioned BALB/c recipients [20]. GFP expression was determined by FCM as surrogate marker for Phl p 5 expression in leukocytes of recipients at different time points throughout the follow-up (weeks 4, 7, 11, 17, and 26; Fig. 2C). Stable and persistent macrochimerism (i.e. chimerism level >1%; *n* = 16 in two experiments) was evident in different lineages of white blood cells (monitored through 25 or 28 weeks after BM transplantation (BMT)). Further chimerism was detectable in spleen, thymus, and BM in FCM at the end of follow-up (data not shown).

### Phl p 5-cyt molecular chimerism leads to a gross reduction of T-cell responses

**Figure 3:**
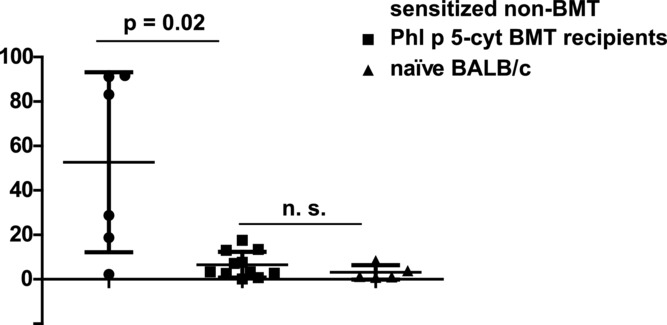
Phl p 5-specific T-cell responses are significantly diminished in Phl p 5-cyt chimeric mice. Phl p 5-specific T-cell proliferation is shown in nontransplanted immunized mice (black circles, *n* = 6), recipients of Phl p 5-cyt-transduced BM (black squares, *n* = 11), and naïve mice (black triangles, *n* = 5) stimulated with Phl p 5. The ability of splenocytes to proliferate was confirmed by unspecific stimulation with Con A (data not shown). Stimulation indices (SI) are shown by Scatter plot (mean ± SD) representing data pooled from two independent experiments. Statistical significance determined by Man–Whitney *U*-test.

Six, nine, and twelve weeks after BM injection, chimeric mice were sensitized with rPhl p 5 and, for specificity control with the major birch pollen allergen rBet v 1. Strong T-cell responses toward Phl p 5 were measured in splenocyte proliferation assays in untreated mice after stimulation with rPhl p 5 [20, 33, 34]. In contrast, chimeric mice showed Phl p 5-specific T-cell unresponsiveness (median SI 7 versus 52 for sensitized non-BMT; Fig. 3). Thus, molecular chimerism with a cytoplasmically expressed allergen induces T-cell hyporesponsiveness.

### Phl p 5-specific IgE and IgG_1_ are diminished in chimeric mice

**Figure 4:**
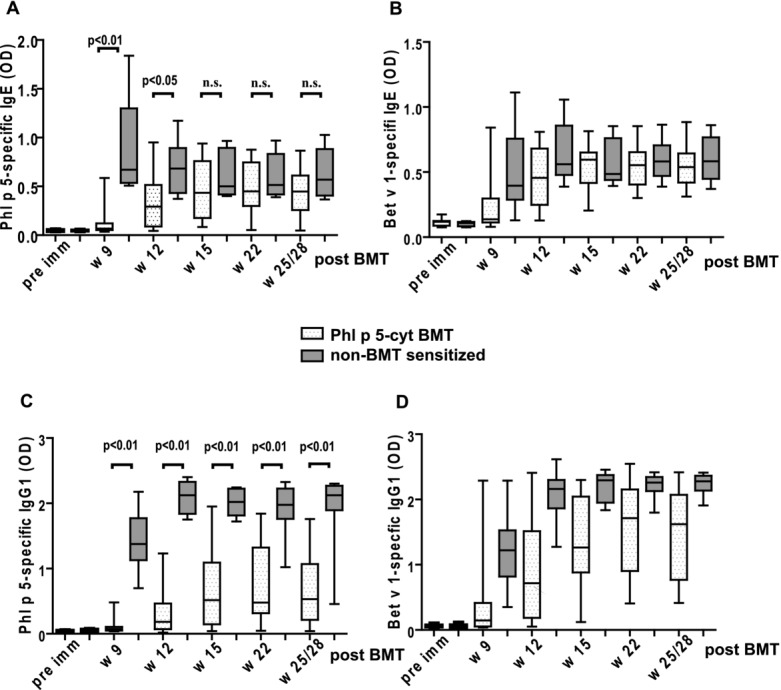
Phl p 5-specific IgE and IgG_1_ is significantly diminished at several time points in Phl p 5-cyt chimeras. (A) The levels of Phl p 5-specific IgE as determined by ELISA are shown at time points indicated and compared between Phl p 5-cyt chimeric mice (*n* = 16) and non-BMT-sensitized mice (*n* = 10). (B) The levels of Bet v 1-specific IgE are shown as in (A). (C) The levels of Phl p 5-specific IgG_1_ as determined by ELISA are shown at time points indicated and compared between Phl p 5-cyt chimeric mice (*n* = 16) and non-BMT-sensitized mice (*n* = 10). (D) The levels of Bet v 1-specific IgG_1_ are shown as in (C). The Ab levels are depicted as box-and-whisker blots representing the mean, interquartile range (box), and SD (whiskers) and data shown are from two independent experiments. Statistical significance determined by Man–Whitney *U*-test.

To investigate if molecular chimerism expressing Phl p 5 cytoplasmically induces tolerance at the B-cell level sera of Phl p 5 chimeric mice (*n* = 16) and non-BMT-sensitized mice (*n* = 10) were analyzed for Phl p 5-specific IgE levels at several time points throughout the whole follow-up of 25 or 28 weeks (Fig. 4A). Levels of Phl p 5-specific IgE were significantly reduced at early time points (weeks 9 and 12). At later time points, Phl p 5-specific IgE levels in sera of chimeric mice were diminished numerically but not significantly compared with those of non-BMT-sensitized control mice. Levels of IgE of the control allergen Bet v 1 were comparable in chimeras and non-BMT controls (Fig. 4B). Notably, Phl p 5-specific IgG_1_ levels were significantly diminished in sera of chimeric mice throughout the follow-up of 28 weeks (Fig. 4C). Hence, although Phl p 5-specific T-cell unresponsiveness was detectable in chimeric mice expressing Phl p 5 cytoplasmically, and although Phl p 5-IgG_1_ levels were significantly reduced, Phl p 5-specific IgE showed only a transient reduction. Thus, T_H_2-dependent B-cell tolerance was incomplete.

### Low levels of Phl p 5-specific T_H_1-dependent IgGs in Phl p 5-cyt chimeric mice

**Figure 5:**
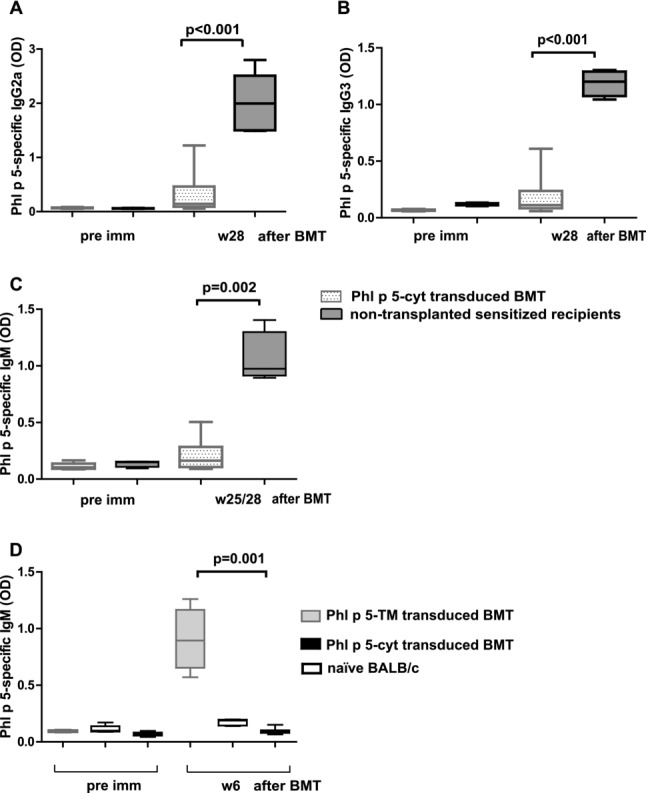
Significant reduction of Th1 isotypes and IgM in Phl p 5-cyt chimeric mice. Phl p 5-specific (A) IgG_2a_, (B) IgG_3_, and (C) IgM levels at late time points of Phl p 5 chimeric mice (*n* = 10) and sensitized mice (*n* = 5) (w28) post-BMT are shown. (D) Phl p 5-specific IgM levels are demonstrated in the sera of chimeric mice expressing membrane-anchored Phl p 5 (Phl p 5-TM transduced BMT; *n* = 5), naïve mice (*n* = 5), and chimeric mice expressing cytoplasmically expressed Phl p 5 (Phl p 5-cyt-transduced BMT, *n* = 16) 6 weeks after BMT. Ab levels are depicted as box-and-whisker blots representing mean, interquartile range (box), and SD (whiskers) and data shown are pooled from two independent experiments. Statistical significance determined by Man–Whitney *U*-test.

To investigate T_H_1-related Ab responses, Phl p 5 isotypes IgG_2a_ and IgG_3_ were measured (Fig. 5A and B). High levels of Bet v 1-specific IgG_2a_ and IgG_3_ were comparable to (Fig. 4 B and D) levels in sera of control mice (data not shown). Phl p 5-specific IgG_2a_ and IgG_3_ were virtually absent even at late time points (28 weeks) after BMT. Thus, interestingly tolerization mechanisms of T_H_1-dependent responses appear to be different from T_H_2-dependent humoral responses in this model.

### Phl p 5-specific IgM

In previous studies, surface expression of allergens in chimeric mice led to the complete absence of specific high affinity isotypes (IgG, IgE, and IgA). However, IgM toward Phl p 5 was detected in the sera of chimeric mice at late time points despite complete T-cell tolerance toward Phl p 5 [20]. The production of IgM toward surface proteins occurs via a T-cell-independent mechanism, while T-cell help is required for induction of IgG [35]. In a related study, Bet v 1-specific IgM in sera of chimeras expressing Bet v 1 (instead of Phl p 5) on the surface of hematopoietic cells was detectable after BMT even before immunization with rBet v 1. Further, the level of IgM did not change even after several immunizations [22]. In the study, herein Phl p 5-specific IgM remained virtually absent in chimeric mice even at late time points (Fig. 5C). In contrast, Bet v 1-specific IgM was detectable in all mice (data not shown). To determine if Phl p 5-specific IgM is T-cell-dependent or T-cell-independent induced, we compared secreted Phl p 5-specific IgM in the sera of mice after BMT before immunizations with allergens 6 weeks after BMT (Fig. 5D). None of the mice transplanted with BM cells that expressed Phl p 5 cytoplasmically — produced IgM (Fig. 5D) in contrast to sera of mice transplanted with the surface expressed allergen.

## Discussion

The studies presented herein demonstrate that molecular chimerism with the major grass pollen allergen Phl p 5 expressed exclusively in the cytoplasm leads to T-cell hyporesponsiveness.

Traditionally, T_H_2 cells have been defined to produce IL-4, IL-5, and IL-13 [36]. T_H_2 cells induce switching of B cells to IgE and IgG_1_ in mice. Interestingly, we found that T_H_2-mediated humoral responses were reduced but not completely avoided. On the other hand, T_H_1-mediated Phl p 5-specific humoral responses such as IgG_2a_ and IgG_3_ were virtually absent in most chimeric mice. Therefore, different mechanisms of tolerance induction such as suppression by regulatory T cells might be sufficient for T_H_1 but not for the T_H_2-mediated B-cell responses.

In some protocols of molecular chimerism, the level of Ag expression was described as an important (although controversial) factor for tolerance induction. While low expression levels of Ag in a molecular chimerism model of hemophilia A led to T-cell hyporesponsiveness, split tolerance (T-cell versus B-cell tolerance) was observed toward the secreted Ag [37]. Contrarily, high-level expression of GFP in syngeneic recipients was described to induce T-cell tolerance after BMT, nevertheless humoral responses were observed. On the other hand, the low-level expression of GFP showed higher engraftment and no specific Ab responses [38]. In our study, the chimerism levels were clearly macrochimeric (i.e. detectable in FCM in different lineages) and quite stable through the whole follow-up suggesting that the Ag levels are rather high when mice received BM with allergen expressed cytoplasmically. Nevertheless, complete B-cell tolerance was achieved when the allergen was expressed on the surface even when chimerism levels were microchimeric (chimerism levels not detectable in FCM at later time points) but persistent [21]. Therefore, the Ag-level seems not to play a major role for the induction of complete B-cell tolerance in this model.

In pilot studies of combined kidney and BMT, mixed chimerism provoked renal allograft acceptance in transplanted individuals [39]. In most patients, renal allograft tolerance without immunosuppression was achieved. Despite T-cell hyporesponsiveness, B-cell tolerance was not complete as several patients developed low levels of HLA class II donor-specific antibodies and/or C4d deposits [40]. Interestingly, these clinical data somewhat resemble with the data in the study herein. Therefore, B-cell tolerance needs special attention in the design of chimerism-based protocols.

Mechanistically, central B-cell tolerance toward surface Ags is induced in the BM by clonal deletion or receptor editing of auto-reactive B cells. Moreover, anergy toward soluble self-Ags is provoked in B cells [33, 41–44]. Further, B-cell tolerance toward intracellularly expressed Ags was suggested to be maintained by the absence of T-cell help [45]. Therefore, low T-cell responses might be responsible for humoral responses toward Phl p 5 expressed intracellulary as a “self-Ag” in this study. T-cell-independent Phl p 5-specific IgM production in chimeric mice varied with the site of Ag expression. While specific IgM was detectable after exposure of Phl p 5 on the surface, IgM was undetectable after administration of BM containing cytoplasmically expressed Phl p 5. Although allergens are T-cell-dependent Ags, some allergens show repeated epitopes and additionally possible posttranslational modifications when expressed in mammalian cells. Possibly, T-cell-independent IgM might be secreted by B1 cells, a distinct B-cell population in mice [46]. B cells can act as highly potent APCs when they endocytose Ags via their specific surface immunoglobulin receptor. While T cells need cell-to-cell contact, B cells are directly activated through the Ag [47, 48].

Therefore, it is likely that in our model B cells are involved in Ag presentation and that surface exposure of allergens is indispensable for induction of complete B-cell tolerance. Surface-expressed allergen peptides or hypoallergenic allergen derivatives devoid of any IgE cross-linking might be ideal candidates in this setting to avoid anaphylaxia and to develop more clinically relevant protocols to avoid IgE-mediated allergy. Although the safety of cell-based therapeutics and the necessary preconditioning of the recipient are still important limitations, cell engineering becomes more and more of interest in different fields of biomedicine and rapid advances in this area might eventually overcome these hurdles [49].

Overall, these data demonstrate that the site of Ag expression is an important factor influencing the type and degree of tolerance achieved by molecular chimerism. Both T-cell and B-cell compartments need to be successfully tolerized to achieve robust tolerance.

## Materials and methods

### Animals

Female BALB/c mice were purchased from Charles River Laboratories (Sulzfeld, Germany). All mice were housed under specific pathogen-free conditions and were used between 6 and 12 weeks of age. All experiments were approved by the local review board of the Medical University of Vienna, and were performed in accordance with national and international guidelines of laboratory animal care.

### Retroviral construct and production of retroviruses

To generate cytoplasmically expressed Phl p 5, full-length Phl p 5 was cloned using the optimized mammalian codon usage (ATG:biosynthetics, Merzhausen, Germany) into the retroviral vector pMMP-IRES-eGFP via XhoI site resulting in pMMP-Phl p 5-IRES eGFP. The sequence was confirmed by double-strand sequencing (VBC-BIOTECH Service GmbH, Vienna, Austria). For virus production, plasmids pMMP-Phl p 5-IRES eGFP, pMD.G, encoding for VSV-G protein and pMLV, encoding for viral proteins gag and pol, were cotransfected using the calcium phosphate method into 293 T cells [50] resulting in VSV-Phl p 5-cyt viruses. Viral supernatants were concentrated by ultracentrifugation (16 500 rpm for 2 h).

### BM transduction and BMT

Mice were transduced as described in [20]. Briefly, BALB/c donors were treated with 5-FU (i.p.) and BMCs isolated cultivated in the presence of cytokines and transduced with VSV-Phl p 5-cyt with an multiplicity of infection (MOI) of 5.

### Immunization of mice with transduced 3T3 cells

The NIH 3T3 mouse fibroblast cell line was cultivated in the presence of DMEM (Gibco, Invitrogen, Oregon, USA) media + 10% FCS and Pen/strep. Cells were transduced with VSV-Phl p 5-TM and VSV-Phl p 5-cyt with an MOI of 10. High expression levels were determined in FCM. A total of 2.5 × 10^6^ cells were injected subcutaneously into naïve BALB/c mice and Phl p 5-specific Ab levels were determined in ELISAs (e.g. 7 weeks) after injection.

### Immunoblot

293 T cells were transduced with VSV-Phl p 5-cyt or VSV-Phl p 5-TMD. Lysates of infected cells and rPhl p 5 (Biomay, Vienna, Austria) were loaded onto a 12% polyacrylamid gel and blotted to nitrocellulose. A Phl p 5-specific rabbit antiserum was diluted 1:1000 in PBS, 0.05% Tween 20, 2% milk powder. Bound rabbit antibodies are detected with anti-rabbit HRP-labeled antibodies diluted 1:2000 in PBS, 0.05% v/v Tween 20, 2% w/v milk powder. Reactive bands were visualized with the SuperSignal West Pico Chemiluminescent Substrate Trial Kit (Pierce, Rockford, IL, USA) and subsequent exposure to CL-X Posure Film (Pierce).

### Immunofluorescence microscopy

Transduced NIH 3T3 cells were grown onto gelatine (1% in PBS) coated cover slips, transduced BMCs were spun onto glass slides. Cells were washed twice with PBS, fixed for 10 min with 3% paraformaldehyde and stained. Cells were permeabilized with Triton X100 solution and antibodies from a Phl p 5-immunized rabbit were used with a dilution of 1:200 stained with fluorochrome-conjugated secondary antibodies (goat α-rabbit IgG Alexa-Fluor 546 nm, Invitrogen). ProLong Gold antifade reagent (Molecular probes, Invitrogen) was used. Stained cells were visualized with the confocal laser scanning microscope LSM 510 Meta (Zeiss, Jena, Germany).

### Flow cytometric analysis

To detect transduced cells among various leukocyte lineages, white blood cells were stained with PE-conjugated antibodies against CD4, CD8, B220, Mac-1, and isotype controls (Abs from Biolegend) and analyzed by FCM. Two-color FCM was used to determine the percentage of GFP-positive cells of particular lineages. The percentage of chimerism was calculated as the net percentage of GFP-positive cells among leukocyte lineages. 3T3 cells transduced with VSV-Phl p 5-TM or VSV-Phl p 5-cyt were trypsinized, regenerated for 2 h in DMEM media, and incubated with Phl p 5-specific rabbit serum (Charles River Laboratories). Cells were incubated with biotinylated anti-rabbit IgG and stained with streptavidin-PE (Abs from Biolegend). A Beckman Coulter flow cytometer (FC500) was used for acquisition, and Beckman Coulter CXP software (for FC500) was used for analysis of flow cytometric data.

### ELISAs

To measure Ag-specific antibodies in the sera of immunized mice, ELISAs were performed as described previously [29]. Plates were coated with the allergens (all obtained from Biomay) rBet v 1 or rPhl p 5 (5 μg/mL), sera were diluted 1:20 for IgE, 1:100 for IgM, IgG_2a_, and IgG_3_, respectively, and 1:500 for IgG1 and bound antibodies were detected with monoclonal rat anti-mouse IgM, IgG_1_, IgE, IgG_2a_, and IgG_3_ antibodies (BD Pharmingen) diluted 1:1000 and a HRP-coupled goat anti-rat antiserum (Biosciences, UK) diluted 1:2000. The substrate for HRP was ABTS (60 mM/L citric acid, 77 mM/L Na_2_HPO_4_ × 2H_2_O_,_ 1.7 mM/L [Sigma-Aldrich, MO, USA], 3 mM/L H_2_O_2_).

### Lymphocyte proliferation assay

Spleens were removed under aseptic conditions and homogenized. Single-cell suspensions were filtered through a 70 μm nylon cell strainer to remove remaining tissue. Erythrocytes were removed by adding cold lysing buffer (Red Blood Cell Lysing Buffer, Sigma-Aldrich). Cells were diluted to a final concentration of 2 × 10^6^ cells/mL and triplicates of 100 μL/well were sowed in 96-well round-bottom plates. Stimulants were added at a concentration of 2 μg/well allergen or as control for proliferation, 0.5 μg/well Con A (Sigma-Aldrich). The plates were incubated at 37°C, 5% CO_2_. On day 4, 0.5 μCi H^3^ thymidine ([methyl-3H], Amersham) per well was added. Sixteen hours later, cells were harvested and thymidine uptake measured in a beta counter (Beta scintillation liquid, Wallac).

### Statistical analysis

The reported *p*-values are results of the Mann–Whitney *U*-test. *p*-Values < 0.05 were considered statistically significant. Error bars indicate SDs. Graph pad prism statistical software (version 5.01) was used for statistical calculations.

## Abbreviations

FCM: flow cytometry
IRES: internal ribosomal entry site

## Supplementary material

As a service to our authors and readers, this journal provides supporting information supplied by the authors. Such materials are peer reviewed and may be re-organized for online delivery, but are not copy-edited or typeset. Technical support issues arising from supporting information (other than missing files) should be addressed to the authors.

## References

[1] Valenta R, Ferreira F, Focke-Tejkl M, Linhart B, Niederberger V, Swoboda I, Vrtala S (2010). From allergen genes to allergy vaccines. Annu. Rev. Immunol.

[2] Larche M, Akdis CA, Valenta R (2006). Immunological mechanisms of allergen-specific immunotherapy. Nat. Rev. Immunol.

[3] Niederberger V, Horak F, Vrtala S, Spitzauer S, Krauth MT, Valent P, Reisinger J (2004). Vaccination with genetically engineered allergens prevents progression of allergic disease. Proc. Natl. Acad. Sci. USA.

[4] Shamji MH, Durham SR (2011). Mechanisms of immunotherapy to aeroallergens. Clin. Exp. Allergy.

[5] Focke M, Swoboda I, Marth K, Valenta R (2010). Developments in allergen-specific immunotherapy: from allergen extracts to allergy vaccines bypassing allergen-specific immunoglobulin E and T cell reactivity. Clin. Exp. Allergy.

[6] Pilat N, Wekerle T (2010). Transplantation tolerance through mixed chimerism. Nat. Rev. Nephrol.

[7] Alexander T, Thiel A, Rosen O, Massenkeil G, Sattler A, Kohler S, Mei H (2009). Depletion of autoreactive immunologic memory followed by autologous hematopoietic stem cell transplantation in patients with refractory SLE induces long-term remission through de novo generation of a juvenile and tolerant immune system. Blood.

[8] Sykes M, Nikolic B (2005). Treatment of severe autoimmune disease by stem-cell transplantation. Nature.

[9] Baranyi U, Gattringer M, Boehm A, Marth K, Focke-Tejkl M, Bohle B, Blatt K (2011). Expression of a major plant allergen as membrane-anchored and secreted protein in human cells with preserved T cell and B cell epitopes. Int. Arch. Allergy Immunol.

[10] Bagley J, Iacomini J (2003). Gene therapy progress and prospects: gene therapy in organ transplantation. Gene Ther.

[11] Alderuccio F, Nasa Z, Chung J, Ko HJ, Chan J, Toh BH (2011). Hematopoietic stem cell gene therapy as a treatment for autoimmune diseases. Mol. Pharm.

[12] Baranyi U, Pilat N, Gattringer M, Wekerle T (2009). A chimerism-based approach to induce tolerance in IgE-mediated allergy. Crit. Rev. Immunol.

[13] Bagley J, Tian C, Sachs DH, Iacomini J (2002). Induction of T-cell tolerance to an MHC class I alloantigen by gene therapy. Blood.

[14] Bracy JL, Iacomini J (2000). Induction of B-cell tolerance by retroviral gene therapy. Blood.

[15] Bracy JL, Sachs DH, Iacomini J (1998). Inhibition of xenoreactive natural antibody production by retroviral gene therapy. Science.

[16] Chan J, Clements W, Field J, Nasa Z, Lock P, Yap F, Toh BH (2006). Transplantation of bone marrow genetically engineered to express proinsulin II protects against autoimmune insulitis in NOD mice. J. Gene Med.

[17] Tian C, Bagley J, Cretin N, Seth N, Wucherpfennig KW, Iacomini J (2004). Prevention of type 1 diabetes by gene therapy. J. Clin. Invest.

[18] Steptoe RJ, Ritchie JM, Harrison LC (2003). Transfer of hematopoietic stem cells encoding autoantigen prevents autoimmune diabetes. J. Clin. Invest.

[19] Chan J, Ban EJ, Chun KH, Wang S, Backstrom BT, Bernard CC, Toh BH (2008). Transplantation of bone marrow transduced to express self-antigen establishes deletional tolerance and permanently remits autoimmune disease. J. Immunol.

[20] Baranyi U, Linhart B, Pilat N, Gattringer M, Bagley J, Muehlbacher F, Iacomini J (2008). Tolerization of a type I allergic immune response through transplantation of genetically modified hematopoietic stem cells. J. Immunol.

[21] Baranyi U, Pilat N, Gattringer M, Linhart B, Klaus C, Schwaiger E, Iacomini J (2012). Persistent molecular microchimerism induces long-term tolerance towards a clinically relevant respiratory allergen. Clin. Exp. Allergy.

[22] Gattringer M, Baranyi U, Pilat N, Hock K, Klaus C, Buchberger E, Ramsey H (2013). Engraftment of retrovirally transduced Bet v 1-GFP expressing bone marrow cells leads to allergen-specific tolerance. Immunobiology.

[23] Huby RD, Dearman RJ, Kimber I (2000). Why are some proteins allergens. Toxicol. Sci.

[24] Dengjel J, Schoor O, Fischer R, Reich M, Kraus M, Muller M, Kreymborg K (2005). Autophagy promotes MHC class II presentation of peptides from intracellular source proteins. Proc. Natl. Acad. Sci. USA.

[25] Neefjes J, Jongsma ML, Paul P, Bakke O (2011). Towards a systems understanding of MHC class I and MHC class II antigen presentation. Nat. Rev. Immunol.

[26] Nimmerjahn F, Milosevic S, Behrends U, Jaffee EM, Pardoll DM, Bornkamm GW, Mautner J (2003). Major histocompatibility complex class II-restricted presentation of a cytosolic antigen by autophagy. Eur. J. Immunol.

[27] Zhou D, Blum J (2004). Presentation of cytosolic antigens via MHC class II molecules. Immunol. Res.

[28] Tian C, Bagley J, Iacomini J (2002). Expression of antigen on mature lymphocytes is required to induce T cell tolerance by gene therapy. J. Immunol.

[29] Li Y, Ma L, Yin D, Shen J, Chong AS (2008). Long-term control of alloreactive B cell responses by the suppression of T cell help. J. Immunol.

[30] Tian C, Bagley J, Kaye J, Iacomini J (2003). Induction of T cell tolerance to a protein expressed in the cytoplasm through retroviral-mediated gene transfer. J. Gene Med.

[31] Andersson G, Denaro M, Johnson K, Morgan P, Sullivan A, Houser S, Patience C (2003). Engraftment of retroviral EGFP-transduced bone marrow in mice prevents rejection of EGFP-transgenic skin grafts. Mol. Ther.

[32] Bagley J, Aboody-Guterman K, Breakefield X, Iacomini J (1998). Long-term expression of the gene encoding green fluorescent protein in murine hematopoietic cells using retroviral gene transfer. Transplantation.

[33] Halverson R, Torres RM, Pelanda R (2004). Receptor editing is the main mechanism of B cell tolerance toward membrane antigens. Nat. Immunol.

[34] Linhart B, Bigenzahn S, Hartl A, Lupinek C, Thalhamer J, Valenta R, Wekerle T (2007). Costimulation blockade inhibits allergic sensitization but does not affect established allergy in a murine model of grass pollen allergy. J. Immunol.

[35] Zinkernagel RM, Cooper S, Chambers J, Lazzarini RA, Hengartner H, Arnheiter H (1990). Virus-induced autoantibody response to a transgenic viral antigen. Nature.

[36] Mosmann TR, Coffman RL (1989). TH1 and TH2 cells: different patterns of lymphokine secretion lead to different functional properties. Annu. Rev. Immunol.

[37] Evans GL, Morgan RA (1998). Genetic induction of immune tolerance to human clotting factor VIII in a mouse model for hemophilia A. Proc. Natl. Acad. Sci. USA.

[38] Eixarch H, Gomez A, Kadar E, George M, Martinez N, Espejo C, Petriz J (2009). Transgene expression levels determine the immunogenicity of transduced hematopoietic grafts in partially myeloablated mice. Mol. Ther.

[39] Kawai T, Cosimi AB, Spitzer TR, Tolkoff-Rubin N, Suthanthiran M, Saidman SL, Shaffer J (2008). HLA-mismatched renal transplantation without maintenance immunosuppression. N. Engl. J. Med.

[40] Porcheray F, Wong W, Saidman SL, De Vito J, Girouard TC, Chittenden M, Shaffer J (2009). B-cell immunity in the context of T-cell tolerance after combined kidney and bone marrow transplantation in humans. Am. J. Transplant.

[41] Goodnow CC, Crosbie J, Adelstein S, Lavoie TB, Smith-Gill SJ, Brink RA, Pritchard-Briscoe H (1988). Altered immunoglobulin expression and functional silencing of self-reactive B lymphocytes in transgenic mice. Nature.

[42] Hartley SB, Crosbie J, Brink R, Kantor AB, Basten A, Goodnow CC (1991). Elimination from peripheral lymphoid tissues of self-reactive B lymphocytes recognizing membrane-bound antigens. Nature.

[43] Gay D, Saunders T, Camper S, Weigert M (1993). Receptor editing: an approach by autoreactive B cells to escape tolerance. J. Exp. Med.

[44] Tiegs SL, Russell DM, Nemazee D (1993). Receptor editing in self-reactive bone marrow B cells. J. Exp. Med.

[45] Taylor JJ, Martinez RJ, Titcombe PJ, Barsness LO, Thomas SR, Zhang N, Katzman SD (2012). Deletion and anergy of polyclonal B cells specific for ubiquitous membrane-bound self-antigen. J. Exp. Med.

[46] Baumgarth N (2011). The double life of a B-1 cell: self-reactivity selects for protective effector functions. Nat. Rev. Immunol.

[47] Rock KL, Benacerraf B, Abbas AK (1984). Antigen presentation by hapten-specific B lymphocytes. I. Role of surface immunoglobulin receptors. J. Exp. Med.

[48] Baumgarth N (2000). A two-phase model of B-cell activation. Immunol. Rev.

[49] Fischbach MA, Bluestone JA, Lim WA (2013). Cell-based therapeutics: the next pillar of medicine. Sci. Transl. Med.

[50] Pear WS, Nolan GP, Scott ML, Baltimore D (1993). Production of high-titer helper-free retroviruses by transient transfection. Proc. Natl. Acad. Sci. USA.

