# Supplementary material for: The site of allergen expression in hematopoietic cells determines the degree and quality of tolerance induced through molecular chimerism
Source: Eur J Immunol. 2013 Jun 14;43(9):2451–60. doi: 10.1002/eji.201243277 (PMC3816328; doi:10.1002/eji.201243277)
Supplement: Supplementary file 1 [file eji0043-2451-SD1.pdf]

Supporting information for

manuscript ID eji.201243277

Ulrike Baranyi, Martina Gattringer, Andreas M. Farkas, Karin Hock, Nina Pilat,
John Iacomini, Rudolf Valenta and Thomas Wekerle

The site of allergen expression in hematopoietic cells determines the degree and quality of tolerance induced through molecular chimerism

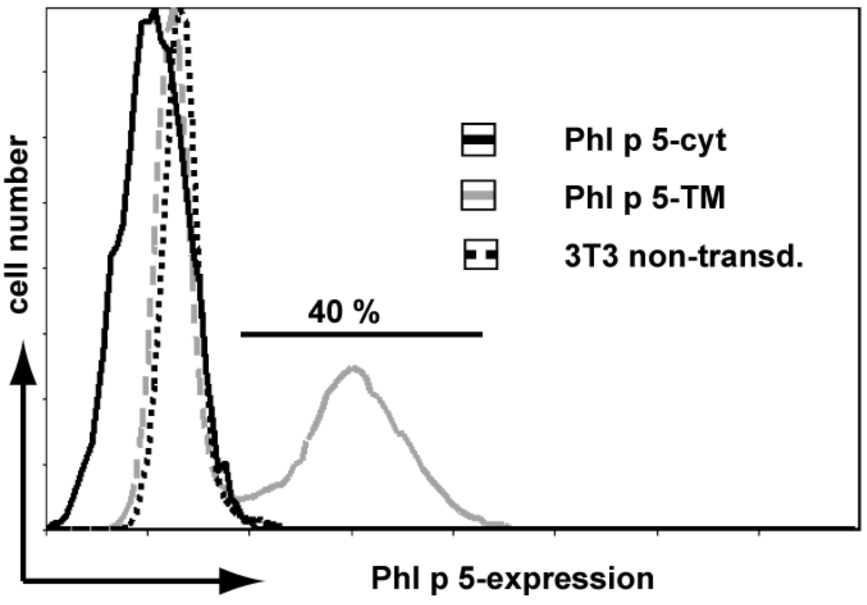

Supporting Information Figure 1: No surface expression of Phl p 5 in Phl p 5-cyt transduced 3T3 cells.

Transduced and non-transduced cells were incubated with Phl p 5-specific antibody, stained with streptavidin PE-conjugates and analyzed by flow cytometry. Histogram shows Phl p 5-cyt transduced and Phl p 5-TM tranduced 3T3 cells and non-transduced 3T3 cells. Data shown are from one representative of three independent experiments.
